# Role of the nervous system in cancer metastasis

**DOI:** 10.3892/ol.2013.1168

**Published:** 2013-01-31

**Authors:** SHA LI, YANLAI SUN, DONGWEI GAO

**Affiliations:** 1 Department of Radiation Oncology, Lanzhou General Hospital of PLA, Lanzhou, Gansu 730050;; 2 Department of Gastrointestinal Tumor Surgery, Shandong Cancer Hospital, Jinan, 250117;; 3 Institute of Oncology, Provincial Hospital Affiliated to Shandong University, Shandong University, Jinan 250021, P.R. China

**Keywords:** nervous system, cancer metastasis

## Abstract

The notion that tumors lack innervation was proposed several years ago. However, nerve fibers are irregulatedly found in some tumor tissues. Their terminals interaction with cancer cells are considered to be neuro-neoplastic synapses. Moreover, neural-related factors, which are important players in the development and activity of the nervous system, have been found in cancer cells. Thus, they establish a direct connection between the nervous system and tumor cells. They modulate the process of metastasis, including degradation of base membranes, cancer cell invasion, migration, extravasation and colonization. Peripheral nerve invasion provides another pathway for the spread of cancer cells when blood and lymphatic metastases are absent, which is based on the interactions between the microenvironments of nerve fibers and tumor cells. The nervous system also modulates angiogenesis, the tumor microenvironment, bone marrow, immune functions and inflammatory pathways to influence metastases. Denervation of the tumor has been reported to enhance cancer metastasis. Stress, social isolation and other emotional factors may increase distant metastasis through releasing hormones from the brain, the hypothalamic-pituitary-adrenal axis and autonomic nervous system. Disruption of circadian rhythms will also promote cancer metastasis through direct and indirect actions of the nervous system. Therefore, the nervous system plays an important role in cancer metastasis.

## Contents

1. Introduction
2. Search strategies and selection criteria
3. Connection, communication and interaction between the neural system and cancer cells
4. Nerve invasion is another route for cancer cell dissemination
5. The nervous system modulates angiogenesis and microenvironments in tumors to affect metastasis
6. Nervous system interacts with immune function and inflammation to influence cancer metastasis
7. Interactions between bone marrow and nervous system: implication for cancer metastasis
8. Cancer as an independent organ is being recognized and should not be isolated from the nervous system
9. Clinical and biological implications for the role of the nervous system in cancer metastasis
10. Conclusions

## 1. Introduction

Since the 1970s, it has been generally accepted in the field of pathology that tumors lack innervation (1). However, the nervous system is a superordinate structure in the body and controls the functions and activities of virtually all other tissues and organs. Cancer tissues are not isolated structures within organisms, therefore they should also interact with the neural system. This speculation has been testified from clinical, epidemiological and experimental studies.

One the one hand, neural functions and tissues exert important functions on cancer initiation and development. Firstly, psychological and behavioral factors, such as chronic stress, depression and social isolation, are considered to contribute to the initiation and progression of certain types of cancer (2–6). Stress is inevitable in human life. The brain is the key organ of the response to stress (7). In the whole brain, activation of the hypothalamic-pituitary-adrenal axis (HPA) plays an important role in the response (2,7). Thus, acting on certain areas in the brain has been reported to influence the growth and development of cancer (8–10). Secondly, the peripheral nervous system is also involved in cancer. 6-Hydroxydopamine was shown to influence the growth rate of certain tumors (11) and induce neuronal changes on the sympathetic nervous system to augment the growth of neuroblastoma (12), suggesting that an intact functional sympathetic nervous system is necessary for certain tumors. Peripheral nerve fibers were found to innervate some tumors (13–16). Neurons of dorsal root ganglia interact with cancer cells (17–19). Denervation of a tumor also alters the behavior of tumor growth (20–22). Peripheral nerve invasion (PNI) induced by cancer is an independent factor for poor prognosis in some cancer patients (23–26).

On the other hand, cancer also has remote effects on neural tissues (27–29). These facts indicate that there is a bidirectional correlation between the nervous system and cancer and challenge the traditional view that cancers lack innervation. Therefore, the role of the nervous system in cancer is gaining attention in cancer research (5, 29–31). Recently, the study by Sloan *et al*(32) study further showed that stress increases distant metastases but has little effect on primary tumor growth, prompting our interest in investigating the role of the nervous system in cancer metastasis.

It is well known that metastasis is the major cause of mortality from solid carcinomas. Despite great advances in metastasis research, the prognosis remains extremely poor. The formation of metastasis is a complex and sequential process, involving four basic steps: i) departure from the primary tumor, ii) survival in the circulatory system, iii) breaching of endothelium and basement membrane of target organs, and iv) establishment of a colony of metastatic cells (33). In these steps, immune functions, inflammation, organic microenvironments and bone marrow are involved (34–40), all of which are directly or indirectly regulated by the nervous system. Thus, cancer metastasis may also establish connections with the nervous system through these pathophysiological changes and functional organs. Therefore, the nervous system may play an essential role in cancer metastasis. To gain a more comprehensive understanding of the role of the nervous system in cancer metastasis, we reviewed English-language literature on this topic and analyzed how the nervous system exerts its functions in cancer metastasis.

## 2. Search strategies and selection criteria

The literature-based review was conducted by searching for keywords in Pubmed and Google Scholar using the search terms: ‘cancer’, ‘tumor’, ‘neoplasm’, ‘malignant’, ‘metastasis’, ‘spread pathway’, ‘stress’, ‘depression’, ‘cancer-related neural disease’, ‘immune’, ‘inflammation’, ‘neuroendocrine’, ‘hypothalamic-pituitary-adrenal axis’, ‘innervation’, ‘nervous system’, ‘neurotransmitters’, ‘neurotrophic factors’, ‘semaphorins’, ‘psychoneuroimmunology’, ‘sympathetic’, ‘vagal’ and ‘vagus’. Only papers published in English prior to March 2012 and focusing on the association between the nervous system and cancer metastasis were included.

## 3. Connection, communication and interaction between the neural system and cancer cells

The nervous system is formed of the central and peripheral nervous systems, which modulate the functions of the whole body through two main methods. The first is that they have a direct connection through a specific structure of classical or non-classical synapses. The other is that they interact with each other through humoral modulations. If the two methods are also found in cancer, the connection between the nervous system and cancer will be established.

Classical and non-classical synapse structures are the elements by which neurons innervate other tissues. Several lines of evidence also support the existence of these anatomical structures in tumor tissues. Nerve fibers have been found in certain tumor tissues, which may be due to i) the possibility of nerve fiber innervation vessels in cancer tissues, ii) the persistence of pre-existing nerves, or nerves becoming included within the cancer owing to the invasive character of its growth, iii) nerve endings growing into cancer tissue, or iv) an integral part of the cancerous growth (41). The first possibility was excluded in the study by Mitchell *et al*(42). Although the second possibility exists and often leads to cancer pain, this type of nerve fiber is surrounded by cancer tissues and hardly exerts any function on cancer cells. The latter two were also observed in previous studies. Nerve fibers in central tumor areas were observed by Seifert *et al*(13,14,43) using a transmission electron microscope. These nerve fibers in tumor tissues were irregularly distributed and had abnormal morphologies, which indicated that occurrences of these abnormal nerve fibers were accompanied with tumor tissues but not the pre-existing nerve fibers in corresponding normal tissues (44). Experimental studies showed that rectal cancer cells stimulated neurogenesis when they were cocultured with neuroepithelial cells (18). Thus, denervation by resection, capsaicin or vagotomy slows tumor growth (20–22) and promotes cancer progression and metastasis (45–47). These facts indicate that neuro-neoplastic synapses may exist between nerve fibers and tumor cells (48,49), and that neurogenesis, like angiogenesis, is also a trait of cancer cells (18,48,50,51). Thus, in primary and pre-metastatic organs, cancer cells actively establish connections with nerve fibers and receive signals from the nervous system.

Although the above studies indicated that tumor tissues were innervated, others do not support this theory (42,52,53). However, humoral modulation is the alternative means for the neural system to modulate other organs, which is also found in cancer. The axes of the systems of the HPA and autonomic nervous system (ANS) are typical pathways of humoral modulation through releasing hormones and neurotransmitters to bind corresponding receptors in other tissues to modulate functions of various tissues, including cancer. Thus, making lesions in these areas have significant effects on the behavior of cancer growth and metastasis. For example, Pollak *et al*(54) showed that hypophysectomy inhibited metastatic behavior in murine osteosarcoma. Function of the pineal gland and effect of spatial disorientation had an important influence on metastasis (55). These effects were mainly via humoral modulators, including neurotransmitters and neuropeptides, neurotrophic factors, semaphorins and other axon growth factors. These modulators of neural development and maintainence have been found to exert essential functions in cancer growth and development, and have been studied and reviewed by numerous authors (56–95). Cancer cells also express these molecules and their receptors. For example, breast cancer cells can express β-endorphin (96), and transplantation of β-endorphin neurons into the hypothalamus inhibit the growth and metastasis of mammary carcinoma (97). Cancer cells also promote neurite formation through generating neutrophic factors and axon growth molecules (98). Thus, they will establish a connection with the nervous system through humoral modulation.

Thus, the existence of neuro-neoplastic synapses and receptors of neural markers in cancer cells provides a substantial basis for communication between neurons and cancer cells. Recent studies have shown that the nervous system influences the process of cancer metastasis through nerve endings and humoral modulations (32,40,44–47,58,67,75–79,85,97,98). For cancer cells to form metastases in ectopic sites, they must depart from the primary tumor and conquer the barriers of primary tissue inhibition. Proteolytic enzymes can help tumor cells escape from primary cancer; i.e. by degrading the surrounding normal tissues. In this process, the overexpression of matrix metalloproteinases (MMPs) in tumor cells is one of the most sustained events (99,100); this may be induced by stress, neural-related factors and neurotransmitters. For example, Wu *et al*(101) reported that stress due to social isolation enhanced invasion and metastasis of colon cancer cells through increasing proteolytic proteases. Yang *et al*(102) also found that stress modulates levels of MMPs through activation of the HPA and sympathetic-adrenal medullary (SAM) axes. Heregulin-β1 and nerve growth factor, as essential factors in the normal development of the nervous system, mediate the activation of MMP-9 and MMP-2 to promote invasion of breast cancer cells (103) and pancreatic cancer cells (104,105). Neurotrophins also promote invasion by enhancing the production of basement membrane-degradative enzymes (106). Norepinephrine (NE) and γ-aminobutyric acid as classical neurotransmitters upregulate the expression of MMPs in nasopharyngeal cancer cells (107) and cancer cells of the prostate (108). Anoikis, a form of apoptosis, results from loss of cell-matrix interactions and acts as a physiological barrier to metastasis (109). Tropomyosin receptor kinase B (TrkB), a receptor of brain-derived neurotrophic factor (BDNF), induces cancer metastasis by suppression of anoikis (110–112). Migration of cancer cells is a prerequisite for metastasis. Axon-guidance molecules, such as slits, semaphorins and netrins, can navigate or inhibit migration of cancer cells (77,85,113–117). The neurotransmitter/receptor system is also involved in cancer cell migration (118). NE, a classical neurotransmitter, has a stimulatory effect on the migration of colon carcinoma cells (119) and breast cancer cells (120). A similar role was also reported for dopamine and neuropeptides, including met-enkephalin, substance P and bombesin (120). γ-aminobutyric acid as an inhibitory neurotransmitter in the brain inhibits the migratory activity of colon carcinoma cells (121). Although these substances are also expressed in other organs (122) and tumor cells, inhibition of the activity of corresponding neurons can reverse their effects on cancer cells. For example, β-blockers acting on the ANS inhibit the migration of cancer cells induced by NE (119). Chemokines exerting important effects on neurogenesis and brain development (123,124) are also important modulators of cancer metastasis (125–127). In the process of cancer cell migration through the blood system, 99.9% of cancer cells are killed, which is called metastatic inefficiency (128). Mechanical forces including shear stress contribute to this inefficiency (129,130) while the shear force is mainly due to vasomotor changes. Nerve endings and receptors of neuropeptides are distributed in vascular walls (131). Based on the anatomical structures, the nervous system modulates the vascular functions, leading to changes in vascular dilation and constriction that can produce mechanical forces. Vascular permeability is important for cancer cell extravasation and colonization. Neuropeptides increase vascular permeability (132,133) to promote cancer cell extravasation and colonization. Thus, the process of cancer cell departure from the primary tumor, invasion, migration, cancer cell inefficiency, extravasation and colonization are associated with the nervous system.

Therefore, the connections between the neural system and cancer through synapse, non-synapse or humoral modulation make it possible to establish reciprocal interactions and communications between cancers and neurons.

## 4. Nerve invasion is another route for cancer cell dissemination

Tumor dissemination from primary cancer is the first step in the formation of metastatic tumors at distant sites. Three major routes are considered to be involved in the spread of tumor cells: lymphatic vessels, blood vessels and serosal surfaces (130). The route of cancer cells along nerve fibers has been a forgotten pathway (134). Recently, PNI has been found in certain types of malignancies and identified to be another pathway for cancer cell dissemination, particularly in the absence of lymphatic or hematogenous metastasis (26). Although PNI has not been considered as a routine test in pathological reports, it has been identified as a key pathological feature in tumors and is used to predict clinical outcomes in many cancers (18,23–26,135–143).

It remains unknown what drives cancer cells to migrate along nerve fibers. In addition, since PNI has been accepted as an emerging route for cancer cell dissemination, PNI requires further definition. For the past 40 years, the predominant theory regarding the pathogenesis of PNI is that distributed tumor cells have the privilege of a low-resistance plane in the neural sheaths, which serves as a conduit for their migration. However, recent studies have shown that reciprocal signaling interactions between tumor cells and nerves may contribute to PNI. To explore these interactions, it is necessary to clarify the process of neurite formation. It has been shown that neurotrophic factors (NGF) and axonal guidance molecules are vital for axonal growth (144–149). These molecules and their corresponding receptors are also found in tumor cells (61,64,84,86,115), which provide the possibility for cancer cells to bind to the neurite (150). In addition, stromal cells, extracellular matrix and their releasing factors are also involved in the process of axonal formation (151), and are also important for tumor cell migration. Thus, these tumor cells spreading along neural fibers may acquire the ability to respond to proinvasive signals within the peripheral nerve milieu and become neurotrophic. It is known that nerve fibers are composed of three layers, the epineurium, perineurium and endoneurium, from the outside to the inside. According to the definition of Liebig *et al*, when tumor cells are found within any of the three layers of the nerve sheath or tumor foci outside of the nerve with involvement of 33% of the nerve's circumference, PNI may be diagnosed (26). This definition provides a new concept in the study of cancer cell dissemination.

## 5. The nervous system modulates angiogenesis and microenvironments in tumors and affects metastasis

Angiogenesis is vital for the development of metastasis (152–154). Vascular endothelial growth factor (VEGF) plays an important role in the process of angiogenesis. Several reports have demonstrated that psychological factors influence tumor angiogenesis in certain types of cancer through regulating of VEGF level. Chronic stress is common in cancer patients and often causes depression and bad moods. It has been reported that chronic stress mediates the vascularization of intraperitoneal metastasis and enhances tumor angiogenesis in the xenograft models of ovarian cancer via increasing VEGF expression (155,156). SNS can be activated in stressed animals (155) and may release neurotransmitters. These neurotransmitters, such as NE, dopamine and bradykinin, have been reported to induce or suppress VEGF expression (132,158–161). Thus, the possible mechanism for chronic stress to enhance tumor angiogenesis may be that neurotransmitters released by activated SNS regulate VEGF expression to promote angiogenesis, which is also applicable to social isolation. It was reported that a perceived lack of social isolation was associated with elevated intratumoral NE in ovarian carcinoma patients. The elevated NE levels were correlated with high grade and advanced stage tumor (162) and indicated metastasis, which may be due to that NE induce VEGF expression and thus lead to stimulate angiogenesis (158). Other neural-related factors also have important effects on tumor angiogenesis. Axon growth molecules such as neuropilins and semaphorins can promote or inhibit angiogenesis (63,86,163–166). A neuropeptide, calcitonin gene-related peptide (CGRP), can facilitate tumor-associated angiogenesis (167). Although it is expressed by other tissues, this experiment testifies that it is derived from neuronal systems. Neuropeptide Y also promotes angiogenesis through regulation of VEGF (168). Circadian rhythm is a basic regulator of normal physiology. Its disruption can also accelerate tumor growth through a Wnt signaling pathway (169), a critical pathway to regulate angiogenesis (170,171). Thus, the neural system is capable of modulating the process of angiogenesis. Moreover, neurogenesis also exists as a trait of certain types of cancer. Cancer cells also express these neural-related factors and their receptors. In fact, there are common molecules between angiogenesis and neurogenesis (172–174), indicating that they may occur in concert in tumors and collectively exert functions on cancer metastasis (175).

The microenvironment regulates not only the growth of primary cancer but also the formation of metastasis, which is formed mainly of stromal cells and signal molecules. On the one hand, these cells and molecules have a direct or indirect correlation with the nervous system (73). Within the tumor microenvironment, stromal cells express β-adrenergic receptors that may be activated by neurotransmitters from local sympathetic nerve fibers and circulating blood. Macrophages in tumor microenvironments are important players in cancer metastasis, and are the key targets of β-adrenergic regulation in several cancer contexts (73). Certain molecules produced and released by neural tissues are the important origins of signals in the tumor microenvironment (176). However, stromal and tumor cells produce neural-related factors to stimulate neurite formation, receive nervous signals and act on the nervous system. Thus, cancer cells are able to take advantage of the factors produced by the nerve fibers to generate a positive microenvironment for cell survival and proliferation in the primary site and secondary organ. Therefore, the microenvironments and neural system establish a feedback loop. They also collectively contribute to the growth of primary cancer and the secondary tumor.

## 6. Nervous system interacts with immune function and inflammation to influence cancer metastasis

The immune system has been identified to play a vital role in cancer metastasis (35,177,178). The association between the immune system and the nervous system has been widely studied and reviewed by many neurobiologists and immunologists (179,180). Several pathways are involved in the interaction between them. Among them, the neuroendocrine and neuronal pathways are the most significant, and are involved in the control of the humoral and cellular immune responses including the immunosuppressive effect, immunosurveillance and immunoenhancement. However, the immune system also influences the central nervous system. The bidirectional neural-immune interactions mainly occur through the neural and immune signal molecules including hormones, neurotransmitters, neuropeptides, cytokines or their receptors, all of which have been demonstrated to contribute to the process of metastasis (181–183). Thus, the neural system modulates cancer metastasis through the immune system. For example, mood disorders, such as stress and depression, inhibit the immune system by decreasing cytotoxic T-cell and natural killer (NK) cells involved in innate immunity that can surveil for cancer metastasis (2). Therefore, stress enhances tumor metastasis via suppression of the immune system (184,185). It also has been identified that mood and relevant immunological status, along with important biological prognostic variables may contribute to the notable outcome variance in early-stage breast cancer (186). Circadian rhythm modulates the immune system by conveying timing information. Circadian disruption may lead to vulnerabilitys to infection and other diseases, including cancer (187,188). Therefore, the nervous system and its psychological or behavioral factors modulate metastasis through immune molecules, cells and effects (189).

The immune effect can induce an inflammatory response. However, unlike the association between the nervous system and immune system, the role of the nervous system in inflammation has only recently been described. It was also found that as well as controlling heart rate and other vital functions in real time, the nervous system reflexively regulates the inflammatory response (190,191). The vagus nerve, the arc of the reflex and neural-related factors such as netrin-1 and neuropeptides, are all involved in the control of inflammation (131,192–200). Thus, the nervous system modulation of cancer development via inflammatory responses has been recognized. Inflammation has been thought to be a driving force for cancer metastasis (36). The inflammatory cells and pathways are the players in cancer metastasis. These players are reported to be influenced by the nervous system. Macrophages are key players not only for inflammation but also metastasis, and are regulated by neuromediators (196,198). Stress has been demonstrated to increase the level of IL-6 (201), which is a proinflammatory factor and plays an important role in cancer metastasis. NE and β-adrenergic receptors that can be induced by stress also regulate IL-6 (202,203). The transcription factor NF-κB is a significant inflammatory factor that has been identified to play a role in cancer development and metastasis (183,204). The neuronal guidance molecule netrin-1 is a direct transcriptional target of NF-κB and demonstrates upregulation under inflammatory conditions (205). Therefore, the nervous system modulates cancer metastasis through immunity and inflammation.

## 7. Interactions between bone marrow and nervous system: implication for cancer metastasis

A recent discovery revealed the mechanism of bone marrow recruiting disseminated tumor cells (206). Moreover, it also plays an important role in sustaining tumor angiogenesis (207), microenvironment (208,209) and formation of the preniche for cancer cells arriving in pre-metastatic organs (210,211). Bone marrow recruits disseminated tumor cells by a recently discovered mechanism (206). It has been found that bone marrow can be innervated by the nervous system, including the ANS and noradrenergic sympathetic nerve fibers (212–216). Preprotachykinin-I (PPT-I) peptides, a family of neuropeptides released by the ANS, are hematopoietic modulators and are highly expressed in cancer cells, which may explain the early integration of cancer cells in the bone marrow (217,218). Progenitor cells of bone marrow may contribute to tumor vascularization (207,219,220). The neurotransmitter dopamine regulates the process of mobilization of endothelial progenitor cells from bone marrow to tumor (221). Stromal cells of the bone marrow are an important source of tumor microenvironments (222), including formation of the pre-metastatic niche in metastatic organs. For example, tumor macrophages, which are derived from bone marrow, contribute to metastasis (223–225). During migration, they are navigated by the nervous system (226–228). Bone marrow stem cells play an important role in the repair of tissue injury, and are also thought to contribute to neurogenesis in cancer (50). They migrate from bone marrow to a terminal, then lodge and mature in the terminal under the control of the nervous system (228,229). Therefore, the role of bone marrow in cancer metastasis is modulated by the nervous system.

## 8. Cancer as an independent organ is being recognized and should not be isolated from the nervous system

As cancer is formed of cancer cells and their surrouding tissues, and there are interactions between tumor cells and their micro- and macroenvironment, cancer is now being considered as a new organ or an independent structure in the body (230). Egeblad *et al*(231) considered tumors to be independent from other organs in the whole organism. This cancer organ is comprised of tumor cells, extracellular matrix, stromal cells and vessels. Although these components are abnormal, they can be organized into a new organ in a pathophysiological manner, which further exchanges and interacts with other organs. Thus, metastasis is viewed to be a result of the interactions between the tumor and the rest of the body. Mareel *et al*(232) viewed cancer as an ecosystem inside a living organism. The ecosystem comprises the primary tumor, lymph node and distant metastasis, bone marrow, blood and lymph circulation. The five elements constitute a vicious circle and interact with each other, finally leading to an influence on the whole system. Egeblad *et al* and Mareel *et al* had different views on cancer but shared the understanding that cancer is an independent system from other organ systems of the body. In this system, the components exchange information and communicate with other systems. However, these two views do not refer to the nerve system. It is well known that the constitutive elements of organs as well as microecosystems are all under the control of nervous system. In the process of metastasis, these elements exchange information with the nervous system. More importantly, the role of the neural system cannot be replaced by other systems. As a central system, it processes and stores information released by cancer cells. In clinical phenomena of cancer metastasis, metastatic tumors still resemble their primary cancers even after decades of dormancy. Comparisons between primary tumors and matched metastasis reveals similarities both at cancer cell and stromal levels, which may be modulated by the neural system.

## 9. Clinical and biological implications for the role of nervous system in cancer metastasis

From the above findings, we can conclude that the neural system has an important influence on cancer metastasis. Stress, depression and social isolation as psychological factors have been reported to promote distant metastasis, which is due to them suppressing immune functions (185), promoting angiogenesis, activating macrophages and releasing proinflammatory factors, such as IL-6 (201) and TNF-α (101) and acting on the HPA and ANS (3,233) to release neurotransmitters (234). Circadian rhythm also influences distant metastasis through modulation of angiogenesis (235), the HPA and the immune system (187,236,237). The brain modulates cancer development including cancer metastasis through the vagus nerve (45). It has significant biological and clinical relevance.

Firstly, stress is a common phenomenon and prompts cancer metastasis, indicating that suppressing stress and modulating mood will be helpful for cancer patients (234,238). Thus, psychologically effective interventions on individuals with a variety of cancers can be resistant to cancer progression and improve clinical outcome for advanced cancer patients (239). Secondly, under circumstances without lymph node and blood metastasis, PNI offers another pathway for the spread of cancer cells. Determining the mechanism of PNI may provide additional options for the prevention and treatment of metastasis by inhibiting the pathway. Thirdly, neoangiogenesis and neurogenesis may exert their effects in concert on cancer metastasis. Inhibition of neoangiogenesis alone has little and even reverse effects on treating metastasis (240). For example, VEGF inhibitors have been reported to have no value in metastatic breast cancer (241) and even enhance metastasis in animal studies (240,242). Establishing the common molecules between the two types of genesis in cancer tissues and identify them as new targets is likely to aid in treating cancer metastasis. Fourthly, modulating the activities of the nervous system has an important influence on cancer metastasis. The brain is able to show functional or pathological changes when other organs in the whole organism are affected by cancer. The monitoring of these changes in the brain may be a new diagnostic approach to detect tumorigenesis and cancer recurrence. The vagus nerve is central in the response to extrinsic and intrinsic stressors and receiving the signals of the brain and other visceral organs, including cancer. Thus, inhibition of the activity of the vagus nerve is another strategy to prevent cancer metastasis (243–245). For example, β-blockers are being considered to be a novel adjuvant to existing therapeutic strategies in clinical oncology (73). Finally, as important modulators in the nervous system and cancer, neurotrophic factors, neuropilins, axonal guidance molecules, neurotransmitters and their receptors are being considered to exploit new drugs to be used to treat metastasis (78,82,111,165,234,246–250).

The study of metastasis is multifaceted (251). Numerous investigators have devoted themselves to metastasis from their own profession. Although their studies have made great advances, a number of questions remain to be addressed. The connection between the neural system and cancer that we propose in this review is not based on analogy of the association between the nervous system and the immune system but on current studies. The aim of this review was to aid individuals to gain a deeper understanding of cancer metastasis to a certain extent. Although it may not produce major breakthroughs in cancer metastsis, the role of the nervous system in cancer metastasis should not be neglected.

## 10. Conclusions

In conclusion, the possible existing synapses in tumor cells and neural-related factors, such as neurotrophic factors and neurotransmitters, make it possible to establish direct connections between the nervous system and cancer. PNI offers another pathway for cancer cell distribution. On an anatomical basis, the nervous system is involved in the processes of metastasis, including tumor cell growth, proliferation, angiogenesis, apoptosis, departure from primary cancer, migration, extravasation and colonization, metastatic inefficiency, and modulators of cancer metastasis such as immunity, inflammation and bone marrow. Therefore, the nervous system plays an important role in cancer metastasis. Understanding its mechanism has important implications for exploring the biology of metastasis and the management of cancer metastasis.
